# Probing structural transformation and optical and magnetic properties in Cr doped GdMnO_3_: Jahn–Teller distortion, photoluminescence and magnetic switching effect

**DOI:** 10.1039/c9ra08562a

**Published:** 2019-12-02

**Authors:** Priyanka Tiwari, Sandeep Kumar, Chandana Rath

**Affiliations:** School of Materials Science and Technology, Indian Institute of Technology (Banaras Hindu University) Varanasi 221005 India crath.mst@iitbhu.ac.in

## Abstract

The systematic evolution of structure, photoluminescence and different magnetic transitions in GdMnO_3_ is reported after Cr doping. With increasing the Cr concentration from 10 to 40 at%, Rietveld refinement of X-ray diffraction patterns demonstrates that an O′ type orthorhombic structure transforms to O type, manifesting a reduction in lattice volume. The noticeable reduction in lattice volume is ascribed to the smaller size of the Cr^3+^ ion compared to Mn^3+^. The structural transformation is accompanied with a considerable decrease in the Jahn–Teller distortion factor evaluated from XRD, Raman and photoluminescence measurements. Magnetic studies reveal a considerable enhancement in Néel temperature (*T*_N_) from ∼42 K for *x* = 0 to 130 K for *x* = 0.4. Interestingly, we observe magnetization reversal (MR) with spin reorientation (TSR) for *x* = 0.3. The mechanism for such a magnetic behavior is discussed on the basis of competition between Mn, Cr and Gd. The incorporation of Cr not only constructively modifies the crystal structure and evokes the magnetic reversal phenomenon but also contributes towards the enhanced emission spectra. The promising structure and magnetic properties of Cr doped GdMnO_3_ offer potential pathways for spintronics and magnetic switching devices.

## Abstract 2

The systematic evolution of structure, photoluminescence and different magnetic transitions in GdMnO_3_ is reported after Cr doping.

## Graphical abstract

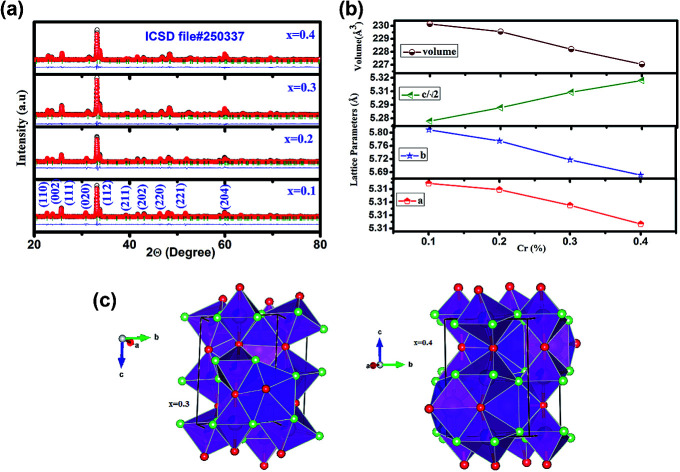

## Introduction

Rare earth manganites (RMnO_3_, R = rare earth) possess complex spin arrangements leading to unusual magnetic ordering such as antiferromagnetic (AFM) and cycloidal spin structures including spin reorientation (*T*_SR_).^1–5^ Since the last decade, a wide group of researchers has reported extensive investigations to comprehend the nature of spin, charge, orbital ordering or the exchange interaction of the transition metals. Recently, theoretical calculations along with experimental evidence reveal that such manganites exhibit distorted perovskites with an orthorhombic structure. The manganites of RMnO_3_ type containing undersized trivalent R ions like GdMnO_3_ demonstrate a ferroelectricity phenomenon induced due to competition between magnetic interactions evoking an antiferromagnetic (AFM) spin ordering that results into the lattice modulations.^6–12^ GdMnO_3_ show intriguing and captivating magnetic properties. GdMnO_3_ crystallizes into an orthorhombic (O′) structure showing canted AFM at ∼23 K along with Néel temperature (*T*_N_) at ∼42 K. In general, the manganites exhibit inherent Jahn–Teller (J–T) distortion evoking unfavorable structural imperfections which essentially modify different physical properties.^13–19^ Usually, the structural distortions can be perceived by employing sensitive characterization tools such as Raman, X-ray absorption, and photoluminescence (PL) spectroscopy *etc.* In this regards, Modi *et al.* have reported that the room temperature Raman spectra of GdMn_1−*x*_Cr_*x*_O_3_ (0 ≤ *x* ≤ 0.2) shows the reduction of Raman shift as doping concentration increases that related to anti-symmetric Jahn–Teller stretching mode and symmetric stretching mode. The broadening of Raman peaks can be consequence of lattice disorder as induced by Cr doping in Mn site.^20^ While Li *et al.* have reported that there is a broadening of the Raman spectrum appeared from 400 to 900 cm^−1^ with increasing magnetic field from 0 to 20 kOe in La_0.75_Ca_0.25_MnO_3_.^21^ The reason behind such broadening is associated with the structural changes. It is known that PL emissions of perovskite materials including CaTiO_3_ and SrZrO_3_ are affected considerably due to the intrinsic structure related distortions.^22,23^ A wide group of researchers have reported the considerable tuning of magnetic properties, in particular after doping various ions at Gd or Mn site.^24^ In this context, Nandy *et al.* and Sarguna *et al.* discuss the significant improvement of the magnetic properties by doping Na^+^ and Y^3+^ in Gd site.^25,26^ The magnetic properties can also be modified if a non J–T active element replaces the J–T active element within the GdMnO_3_ lattice. In this regard, including our previous studies,^24^ Pal *et al.* and Modi *et al.* have reported the magnetic properties of GdMnO_3_ after doping Fe and Cr at Mn site, respectively.^20,27^ While Pal *et al.* report the presence of *T*_SR_ in GdMn_1−*x*_Fe_*x*_O_3_ when *x* > 0.4, we could show in our previous report that *T*_SR_ could observe even when *x* = 0.3.^24^

Since Cr exhibits desired non J–T active characteristic, the interaction between R and Cr^3+^ also suggests an unusual negative magnetization (NM) phenomenon.^28^ The unexpected magnetic behavior of NM was first predicted by Néel in the year, 1948.^29^ In case of NM behavior, a definite temperature at which the value of magnetization becomes zero is termed as the compensation temperature (*T*_comp_). Interestingly, the magnetization shows negative value along with some spin reorientation below *T*_comp_. In general, the NM phenomenon emerges either with changing the temperature or the degree of the magnetic field. Therefore, such a phenomenon is unusual since it arises even under the influence of applied magnetic field having fix direction. The exclusive properties of the NM have laid down various potential applications such as magnetic switching, magneto caloric and spintronics devices *etc.*^30–32^ Usually, NM phenomenon has been reported in rare earth orthochromites, orthoferrites, and orthovanadates.^33–37^ For example, Pena *et al*. have found negative magnetization in Gd_1−*x*_Ca_*x*_MnO_3_ (0.2 ≤ *x* ≤ 0.4) powders with ferrimagnetic transition.^10^ Yingnan *et al.* report that when Sr is doped at Gd site, the magnetization reversal is observed in Gd_1−*x*_Sr_*x*_MnO_3_ (0.1 ≤ *x* ≤ 0.3).^38^ In epitaxial film of Gd_0.67_Ca_0.33_MnO_3_, Ma *et al.* show the NM behavior at low temperature due to f–d interaction.^39^ Modi *et al.* have reported the structural, electrical and magnetic properties of GdMn_1−*x*_Cr_*x*_O_3_ when *x* ≤ 0.2 having orthorhombic structure with space group *Pbnm* synthesized through solid state reaction technique. It has been observed a crossover of ZFC magnetization from positive value at *x* = 0 to negative value at *x* = 0.2. The negative magnetization is understood on the basis of competing WFM–FM interactions.^20^ It is interesting to extend the Cr doping concentration above 0.2 to examine the unusual magnetic behavior in GdMnO_3_. Besides, Cr^3+^ also plays an important role in negative magnetization and provides a prospective pathway to realize the magneto electric coupling and enhancement in multiferrocity. Surprisingly, there exists only a few reports which discuss NM phenomenon in rare earth manganites.

Therefore, in this work, we demonstrate the correlation between structural transformation and intriguing magnetic transitions in Cr doped GdMnO_3_ varying dopant concentration from 10 to 40 at%. GdMn_1−*x*_Cr_*x*_O_3_ (*x* = 0.1, 0.2, 0.3 and 0.4) synthesized *via* facile sol–gel method reveals that O′ type orthorhombic structure transforms to O type followed by a notable decrease in the J–T distortion factor with increasing Cr concentration. Raman and PL spectra endorse the dramatic decrease in the J–T distortion factor. Temperature and field dependent magnetization including time dependent remanent magnetization measurements are carried out to evaluate unique properties like NM, compensation, magnetic switching *etc.* in these compounds.

## Experimental details

GdMn_1−*x*_Cr_*x*_O_3_ (*x* = 0.1, 0.2, 0.3 and 0.4) were prepared *via* facile and robust sol–gel technique. For Cr doped GdMnO_3_ sample, the stoichiometric amounts of gadolinium nitrate (Gd(NO_3_)_3_·6H_2_O, Sigma Aldrich, >99.9%), manganese chloride (MnCl_2_·6H_2_O, Himedia, >98%) and chromium chloride (CrCl_3_·6H_2_O) were mixed with distilled water and citric acid. The ratio of cation to citric acid was kept constant at 1 : 1. First, the solution mixture was continuously stirred at 80 °C for 20 minutes. Then, the dropwise addition of ethylene glycol to the mixture solution formed the gel. Afterwards, the obtained gel was dried at 100 °C for 6 hours to produce precursor resin. Following grinding of resin using mortar and pestle, the powders were procured which appeared dark brown in color. GdMn_1−*x*_Cr_*x*_O_3_ (*x* = 0.1, 0.2, 0.3 and 0.4) samples were calcined at 1100 °C for 5 hours in air. For structural characterization, the powders were studied using Rigaku make powder X-ray diffractometer (XRD) operating in the Bragg Brentano geometry equipped with a 3 kW rotating anode producing Cu K_α_ radiation. The prominent vibrational modes emerging in GdMn_1−*x*_Cr_*x*_O_3_ were investigated through Raman spectroscopy using Horiba Jobin Yvon. The excitation and emission spectra were collected with PL spectrometer (Hitachi F-4600). The magnetic property measurement system (MPMS) of Quantum Design, USA working between temperature range from 2 to 300 K were employed to characterize the magnetic properties.

## Results and discussion

**Fig. 1:**
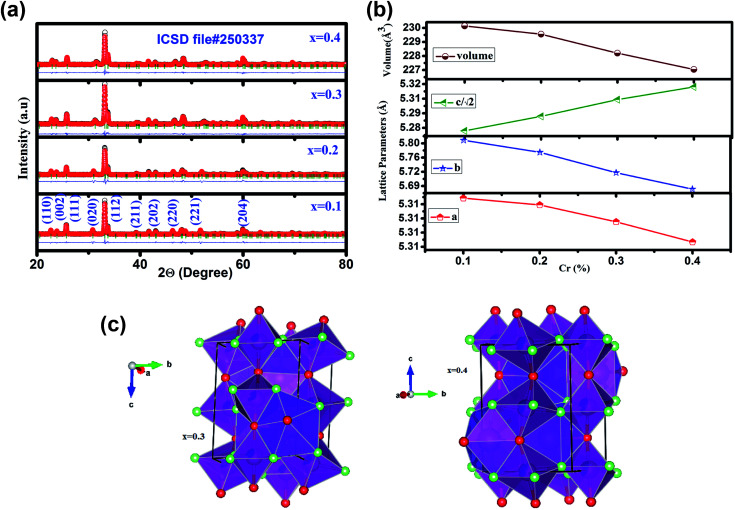
(a) X-ray diffraction (XRD) patterns of GdMn_1−*x*_Cr_*x*_O_3_ (*x* = 0.1, 0.2, 0.3 and 0.4) powders calcined at 1100 °C fitted using Rietveld refinement of Fullprof program. The observed and refined XRD patterns are shown by black and red dots, respectively. The difference between observed and calculated patterns (*I*_o_ − *I*_c_) are shown by blue line at the bottom of the respective one. The tick marks above the difference patterns represent the position of the Bragg peaks. (b) Variations in lattice parameters and volume with Cr concentration. The errors are smaller than the point size. (c) Crystal structure obtained from Rietveld refinement drawn using Vesta software for *x* = 0.3 and *x* = 0.4. Purple, pink, red and green solid balls are denote Gd, Mn, O1 and O2 atoms, respectively.

Fig. 1 (a) illustrates the room temperature XRD patterns along with Rietveld refinement of GdMn_1−*x*_Cr_*x*_O_3_ (*x* = 0.1, 0.2, 0.3 and 0.4). XRD data refined over 2*Θ* = 20–80° having O′ type distorted perovskite structure considering pseudo-Voigt function, space group, *Pbnm*, upto *x* = 0.3 as evidenced from the Bragg's positions indicated by green bars. Thus, GdMn_1−*x*_Cr_*x*_O_3_ crystallizes as a single phase without any trace of impurity phase. Interestingly, with increasing concentration of Cr from 0.3 to 0.4, O′ orthorhombic structure transforms into O type one with space group *Pbnm*. In our recent work, we have demonstrated structural transformation from O′ orthorhombic to O type orthorhombic one by varying concentration of Fe from 30 to 50 at% in GdMnO_3_.^24^ Due to orthorhombic structure, we have expressed the lattice parameters as *a* ≈ *b* ≈ *c*/√2. For *a* < *c*/√2 < *b*, the perovskite adopts O type orthorhombic structure whereas the condition *i.e. c*/√2 < *a* < *b* satisfies the O′ orthorhombic structure. After Rietveld refinement, the obtained parameters are tabulated in Table 1. Following the fitting parameters, the Rietveld refinement appears to be fairly satisfactory looking at the difference plot shown as blue line and the small value of *χ*^2^. The change in lattice parameters with composition of Cr is shown in Fig. 1(b). One may notice from Fig. 1(b) that with increasing Cr concentration while *a* and *b* decreases, *c* increases with reduction in lattice volume from 231.22 to 228.20 Å^3^ with increasing Cr from 0.1 to 0.4. The significant decrease in lattice volume is attributed to the difference in ionic radii of Cr and Mn ions. The smaller ionic radius of Cr^3+^ (0.61 Å) than that of Mn^3+^ (0.64 Å) shows a noticeable decrease in lattice volume confirming the substitution of Cr^3+^ ions at Mn^3+^ site in the lattice. Further, on the basis of parameters obtained from Rietveld refinement, we show a representative unit cell of the crystal structure in three dimension (3D) indicating MnO_6_ octahedra with spatial orientations and atom positions using Vesta software depicted in Fig. 1(c) for *x* = 0.3 and 0.4.^40^ In MnO_6_ octahedra, O1 atoms (red balls) reside at the two apical positions whereas O2 atoms (green balls) occupy four equatorial positions. The equatorial Mn–O2 bonds have two distinct bond lengths indicated as long (*l*) and short (*s*). If all of the Mn–O and average 〈Mn–O〉 bond lengths are calculated from XRD patterns, the coherent J–T distortion is estimated by following expression.

1 *σ*_JT_^2^ = 1/3Σ_*i*_[(Mn–O)_*i*_ − 〈Mn–O〉]^2^

**Table 1:** Structural parameters of GdMn_1−*x*_Cr_*x*_O_3_ (*x* = 0.1, 0.2, 0.3 and 0.4) nanoparticles at room temperature revealed from the structure refinement

Parameters	*x* = 0.1	*x* = 0.2	*x* = 0.3	*x* = 0.4
Gd (*x*, *y*, *z*)	(0.9833(6), 0.0806(4), 0.25)	(0.9840(7), 0.0768(5), 0.25)	(0.9850(5), 0.0720(3), 0.25)	(0.9807(5), 00.691, 0.25)
Mn/Cr (*x*, *y*, *z*)	0.5, 0, 0	(0.5, 0, 0)	(0.5, 0, 0)	(0.5, 0, 0)
O1 (*x*, *y*, *z*)	(0.095(3), 0.492(3), 0.25)	(0.096(4), 0.475(4), 0.25)	(0.1137(3), 0.4649(3), 0.25)	0.087(4), 0.450(4), 0.25
O2 (*x*, *y*, *z*)	(0.703(3), 0.303(3), 0.0437(19))	(0.705(4), 0.298(4), 0.047(2))	(0.7788(3), 0.8215(3), 0.0495(3))	0.697(3), 0.306(3), 0.035(2)
*χ* ^2^	1.33	2.12	2.77	2.30
Mn–O1 (Å)	1.93	1.944(6)	1.982(9)	1.958(6)
Mn–O2 (Å) (*l*)	2.24	2.07(2)	2.214(14)	2.046(17)
Mn–O2′ (Å) (*s*)	1.87	1.99(2)	1.901(7)	1.968(16)
Mn–O1–Mn (deg)	148	148.4	142.53(12)	147.8(2)
Mn–O2–Mn (deg)	147.5	151.1(9)	148.86(11)	151.2(7)
|*a* − *b*|	0.4896	0.4594	0.4089	0.3685
*σ*_JT_	0.066	0.053	0.046	0.037

The estimated values of *σ*_JT_ tabulated in Table 1. For GdMnO_3_, there exists a large J–T distortion factor *i.e. σ*_JT_ = 0.2.^24^ However, after incorporating Cr into the host lattice, *σ*_JT_ is found to be ∼0.066 which reduces to 0.053 and 0.046 with increasing the Cr concentration from 0.1 to 0.2 and 0.3, respectively. For *x* = 0.4, where O′ to O orthorhombic structural transformation takes place, J–T distortion reduces to 0.037 which is almost 50% of the J–T factor observed in case of *x* = 0.1. The considerable reduction in J–T distortion factor is ascribed to the replacement of J–T active element Mn^3+^ ions by the non-J–T active, Cr^3+^ ions in the lattice. Further, we observe that the difference in *a* and *b i.e.*, |*a* − *b*| deceases from 0.4896 to 0.3685 when Cr concentration increases from 10 to 40 at%. Such decreasing trend clearly indicates that orthorhombic structure tends towards a more symmetrical structure *i.e.*, the tetragonal structure.

**Fig. 2:**
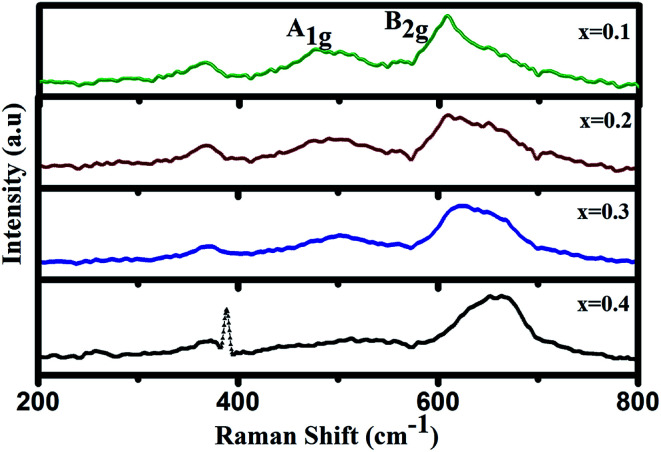
Raman spectra of GdMn_1−*x*_Cr_*x*_O_3_ (*x* = 0.1, 0.2, 0.3 and 0.4).

To confirm the change in J–T distortion with increasing Cr concentration, we have undertaken of Raman spectroscopic measurement. Raman spectra of GdMn_1−*x*_Cr_*x*_O_3_ (*x* = 0.1, 0.2, 0.3 and 0.4) at room temperature are shown in Fig. 2. In case of *x* = 0.1, the spectrum exhibits a tilting mode at 365 cm^−1^ along with the two J–T stretching modes *i.e.* anti-stretching (as) mode, A_1g_ at 474 cm^−1^ and stretching (s) mode, B_2g_ at 608 cm^−1^ in Fig. 2.^41^ A_1g_ and B_2g_ modes arise due to in-plane antisymmetric vibration and stretching of O2, respectively which are correlated with J–T distortion. With increasing concentration of Cr from 0.2 to 0.3, both A_1g_ and B_2g_ are found to be red-shifted indicating smaller Mn–O bond length (*d*_Mn–O_) keeping the intensity of peaks almost same. Apparently, at *x* = 0.4, the peak intensity drastically suppressed. Similar reduction in the peak intensity has been reported in GdMnO_3_ after increasing the Cr concentration to 0.2 by Modi *et al.*^20^ Li *et al.* have also found the broadening of the Raman peaks within 400–900 cm^−1^ in La_0.75_Ca_0.25_MnO_3_ when the magnetic field increases from 0 to 2.5 kOe at room temperature. Such broadening of peaks indicates the reduction in orthorhombic distortion or MnO_6_ octahedral distortion inducing structural changes.^21^ In the present case, the drastic reduction in peak intensity in *x* = 0.4 thus confirms the structural transformation from O′ to O type orthorhombic leading to significant decrease in J–T distortion. The fingerprint of J–T distortion induced structural transformation in Cr doped GdMnO_3_ is investigated further through photoluminescence studies.

### Photoluminescence properties

**Fig. 3:**
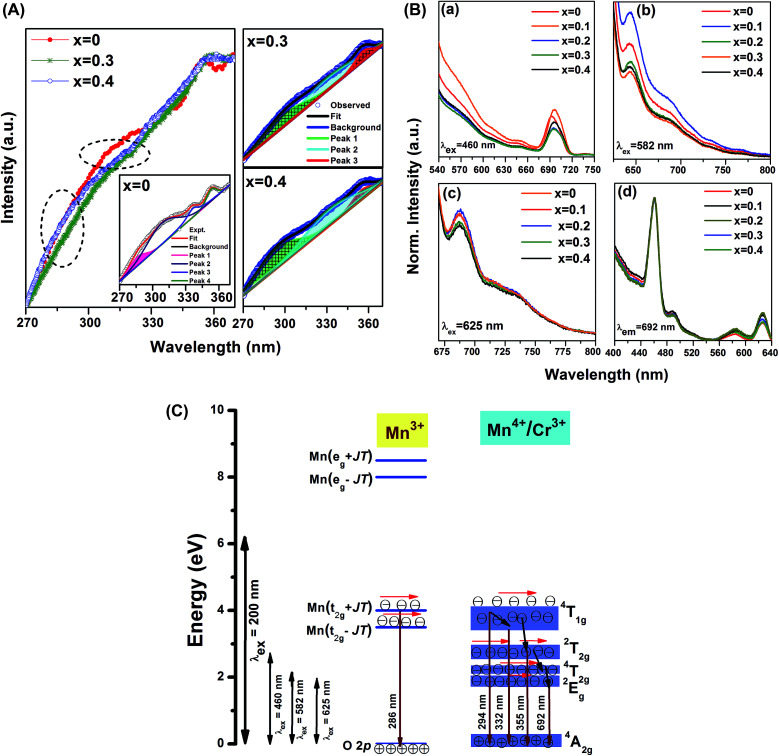
(A) The emission spectra of GdMn_1−*x*_Cr_*x*_O_3_ (*x* = 0, 0.3 and 0.4) under the excitation wavelength of 200 nm (Inset depicts the deconvoluted peaks). (B) The emission spectra of GdMn_1−*x*_Cr_*x*_O_3_ (*x* = 0, 0.1, 0.2, 0.3 and 0.4) at (a) *λ*_ex_ = 460 nm, (b) *λ*_ex_ = 582 nm, (c) *λ*_ex_ = 625 nm and (d) the excitation spectra after monitoring *λ*_em_ = 692 nm. (C) The proposed energy band diagram for Cr doped GdMnO_3_.

Fig. 3(A) depicts the emission spectra of GdMn_1−*x*_Cr_*x*_O_3_ (*x* = 0, 0.3 and 0.4) after exciting with 200 nm. The spectra consist of broad emission peaks in wavelength range of 270–370 nm. For a simplified comparative analysis, we have deconvoluted the emission spectra to reveal dominant emission peaks. The emission spectrum of pure GdMnO_3_ shows four distinct peaks at ∼286, 308, 336 and 353 nm. In case of large J–T distortion, it is known that Mn^3+^ exhibits four distinct energy levels at 3.5, 4, 8 and 8.5 eV corresponding to transition from O 2p to Mn(t_2g_ − JT), Mn(t_2g_ + JT), Mn(e_g_ − JT), Mn(e_g_ + JT), respectively.^42^ The emission peak at ∼286 can be assigned to O 2p → Mn(t_2g_ + JT) transition of Mn^3+^. The peak at 308 nm is attributed to the band transition, ^4^T_1g_ → ^4^A_2g_ of Mn^4+^. Owing to broad nature of this peak, the energy level Mn(t_2g_ − JT) of Mn^3+^ having comparable energy to Mn^4+^ can also contribute towards this emission. The other peak located at 336 nm while emerges from relaxation of electrons from ^4^T_1g_ energy band to ^4^A_2g_, peak at 353 nm originates from the electronic transition, ^2^T_2g_ → ^4^A_2g_, corresponding to Mn^4+^.^43^ After doping Cr (*x* = 0.3 and 0.4), three peaks are found to be located at ∼294, 332 and 355 nm. Surprisingly, the emission peak at ∼286 nm observed in GdMnO_3_ disappears completely by incorporating Cr in host lattice. The disappearance of above peak could be due to the J–T distortion factor of 0.2 observed in GdMnO_3_ which reduces to 0.046 and 0.037 in *x* = 0.3 and 0.4, respectively. Under reduced J–T distortion, the energy gap between Mn(t_2g_ − JT) and Mn(t_2g_ + JT) reduces significantly. Considering this fact, the broad emission peak at ∼294 nm arises mainly from ^4^T_1g_ → ^4^A_2g_ of Mn^4+^ whereas other peaks at ∼332 and 355 nm remain same. In this context, in our previous work, we have also shown the disappearance of peak at ∼286 nm after doping Fe in GdMnO_3_ where J–T distortion has been reduced by one order magnitude.^24^ Moreira *et al.* have also mentioned the disappearance of PL emission peak in CaTiO_3_ due to undistorted or ordered TiO_6_ clusters.^23^ J–T distortion factor less by one order magnitude thus influences the PL spectra in Cr doped GdMnO_3_.

Fig. 3(B) shows the emission spectra of GdMn_1−*x*_Cr_*x*_O_3_ (*x* = 0–0.4) under an excitation wavelength of 460, 582 and 625 nm. After exciting at wavelength of 460 nm, the emission spectrum of GdMnO_3_ exhibits a broad peak ranging from 675–720 nm centered at ∼692 nm lying in red region. This emission peak is attributed to the spin-forbidden, ^2^E_g_ → ^4^A_2g_ transition of Mn^4+^ ion. Since Mn^4+^ exhibits large effective positive charge, this transition is found to be dominated in the emission spectrum due to strong crystal field of the host.^43^ After incorporating Cr into GdMnO_3_, although the intensity of emission peak at ∼692 nm changes significantly, no change in peak position is observed. Exciting GdMnO_3_ with 582 nm, one broad emission having a maximum at 645 nm is observed indicating a blue-shift of ∼45 nm. Under an excitation wavelength of 625 nm, for GdMnO_3_, the broadband emission peak is found to be located at ∼690 nm. In case of *x* = 0–0.2, the intensity of peak is nearly same which diminishes for *x* = 0.3 and 0.4. The peak position of emission *i.e.* ∼692 nm of Mn^4+^ can vary significantly depending on the host material and excitation wavelength. For example, while this peak is observed at ∼617 nm in Na_2_SiF_6_, same peak is found to be red shifted by ∼100 nm showing prominent emission at 723 nm in SrTiO_3_.^44^

The excitation spectra have been taken by monitoring *λ*_em_ = 692 nm depicted in Fig. 3(C). In GdMnO_3_, the excitation spectrum is comprised of a strong peak at ∼460 nm along with a shoulder peak at ∼490 nm. This excitation peak is primarily attributed to ^4^A_2g_ → ^2^T_2g_ electronic transition of Mn^4+^ ion in the lattice. Two more sharp peaks are observed to be centered at ∼582 and ∼625 nm. The former one originates because of ^4^A_2g_ → ^4^T_2g_ transitions whereas the latter one is ascribed to the local vibration-activated ^2^E_g_ ↔ ^4^A_2g_ transitions of Mn^4+^ ion.^44^ We do not observe any change in the intensity of peak at ∼460 nm with increasing Cr concentration. However, one can see an appreciable enhancement in the intensity of excitation peaks at ∼582 and 625 nm for *x* = 0.1 and 0.2 which reduces when *x* = 0.3 and 0.4. It is known that energy bands of Cr^3+^ are almost similar to Mn^4+^ which may result into overlapping of different energy bands. Therefore, we have proposed an energy band diagram to explain the luminescence behavior of GdMn_1−*x*_Cr_*x*_O_3_ showing various energy levels such as O 2p, Mn(t_2g_ − JT), Mn(t_2g_ + JT), Mn(e_g_ − JT), Mn(e_g_ + JT) of Mn^3+^ under J–T effect, ^2^E_g_, ^4^T_2g_, ^2^T_2g_, ^4^T_1g_, ^4^A_2g_ associated to Mn^4+^ and Cr^3+^ ion, respectively. After exciting with 200 nm, the excited electrons do not reach to Mn(e_g_ − JT) and Mn(e_g_ + JT) of Mn^3+^ due to large energy gap. However, the energetic electrons are only transferred to Mn(t_2g_ − JT) and Mn(t_2g_ + JT) which relax to O 2p showing emissions at 286, 294/308, 332 and 355 nm. After exciting with 460 nm, the electrons are not excited to t_2g_ of Mn^3+^ or ^4^T_1g_ of Mn^4+^/Cr^3+^ due to significant energy gap. In pure GdMnO_3_, the excited electrons reach at ^2^T_2g_ of Mn^4+^ which follow multistep relaxation *via*^4^T_2g_ to ^2^E_g_ and eventually come back to ^4^A_2g_ inducing strong emission peak at ∼692 nm. In the presence of Cr^3+^ ion, the similar energy levels of Cr^3+^ and Mn^4+^ can influence the intensity of this emission peak by providing additional energetic electrons. Under excitation with higher wavelength of 582 nm, the excited electrons residing at ^4^T_2g_ of Mn^4+^/Cr^3+^ de-excited to ^2^E_g_ and finally relax to ^4^A_2g_ showing a similar emission band at ∼645 nm. However, at *λ*_ex_ = 625 nm, this emission band arises due to absorption/emission process occurring between ^4^A_2g_ and ^2^E_g_ energy levels. Thus, it is established that in Cr doped GdMnO_3_, the photoluminescence properties of Mn^4+^ can be modified by Cr^3+^ ion due to the presence of additional energetic electrons providing improved emissions in red region under different excitation wavelengths.

### Magnetic properties

**Fig. 4:**
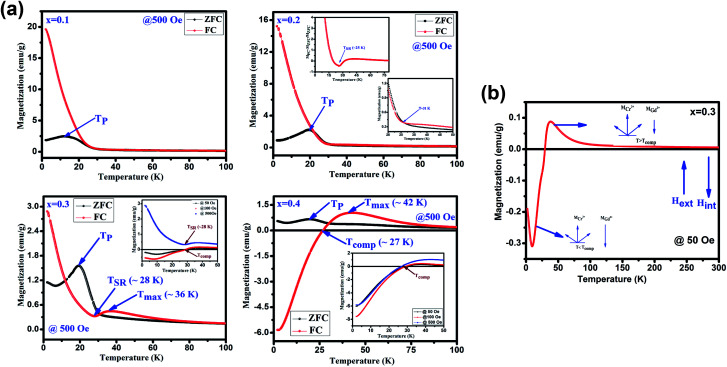
(a) Temperature dependent magnetization under zero field cooling (ZFC) and field cooling (FC) measured at 500 for GdMn_1−*x*_Cr_*x*_O_3_ (*x* = 0.1, 0.2, 0.3 and 0.4) samples. Insets show the FC at different field. (b) Schematic diagrams of the Cr^3+^ moment (*M*_Cr_) and Gd^3+^ moments under different temperature range.

Fig. 4(a) depicts the temperature dependent magnetization (*M vs. T*) under the applied magnetic field of 500 Oe for GdMn_1−*x*_Cr_*x*_O_3_ (*x* = 0.1–0.4). With decreasing temperature from 300 K, below *T*_N_, *M*_ZFC_ and *M*_FC_ bifurcates at ∼31 K for *x* = 0.1. Further, decreasing temperature, while *M*_ZFC_ increases and attains the maximum magnetization at temperature *T*_P_, which is broad in nature, *M*_FC_ increases continuously. In the case of *x* = 0.2, however, after bifurcation of *M*_ZFC_ and *M*_FC_ at ∼63 K, *M*_ZFC_ crosses over the *M*_FC_ at temperature ∼31 K, indicating a magnetic phase transition. Upon reducing temperature further, *M*_ZFC_ attains maxima (∼2.2 emu g^−1^) at *T*_P_ ∼ 19 K, whereas *M*_FC_ increases continuously. For *x* = 0.3, although bifurcation is observed at ∼95 K, *T*_P_ remains same as in case of *x* = 0.2 except a decrease in *M*_max_ to ∼1.56 emu g^−1^. On the other hand, *M*_FC_ in the case of *x* = 0.3 shows a maximum of 0.45 emu g^−1^ at *T*_max_ ∼36 K followed by decrease in magnetization attaining a minimum value of ∼0.32 emu g^−1^ at ∼28 K. The temperature at which minimum magnetization observed is known to be spin-reorientation temperature (*T*_SR_). Although *T*_SR,_ is not detectable from ZFC and FC plot in *x* = 0.2, it is observed at ∼25 K by plotting (*M*_ZFC_ − *M*_FC_)/*M*_ZFC_*vs. T* (shown in the inset Fig. 4(a)). In case of *x* = 0.4, after bifurcation of *M*_ZFC_ and *M*_FC_ at ∼110 K, *M*_ZFC_ attains a maxima of 0.71 emu g^−1^ at ∼19 K same as the *T*_P_ of *x* = 0.2. Besides, with decreasing temperature, *M*_FC_ increases showing a *M*_max_ ∼1 emu g^−1^ at ∼42 K and further decreasing temperature, *M*_FC_ crosses the temperature axis (*T* = 0) at ∼27 K, known to be compensation temperature, *T*_comp_. Below *T*_comp_, the *M*_FC_ becomes negative and attains a minimum magnetization, −5.83 emu g^−1^, at temperature ∼2 K. It is important to note that when *x* is 0.4, an interesting property of magnetization reversal is observed with applying magnetic field of 500 Oe. Further, decreasing the applied magnetic field to 50 and 100 Oe, we could observe the magnetization reversal in *x* = 0.3 as well which is absent under an applied field of 500 Oe. Moreover, except at *x* = 0.1, we show that with increase in Cr concentration upto 0.4, one can observe *T*_SR_ and *T*_comp_ with varying the applied field from 50 to 500 Oe. The mechanism of the magnetization reversal can be understood in terms of negative exchange interaction between two components such as Cr and Gd ions. The ferromagnetic order emerging due to canted AFM ordering of Cr^3+^ produces an internal field at paramagnetically ordered Gd^3+^ ions which are aligned to that of net Cr^3+^ moment. When external field is applied, the component of Cr^3+^ (*M*_Cr_) is pointed along the external field direction while Gd^3+^ ion experiencing the internal field opposite to that of external field. Therefore, the net magnetization of the system, *M*_s_ = *M*_Cr_ − *M*_Gd_ varies with temperature and external field. In the region *T* > *T*_comp_, the *M*_Cr_ dominates over *M*_Gd_ resulting into a net positive magnetization in field direction giving a maxima in *M*_FC_ (shown in Fig. 4(b)). At a critical temperature magnetization corresponding to *M*_Gd_ and *M*_Cr_ cancel out, resulting a zero magnetization known as *T*_comp_. Decreasing the temperature further the magnetization becomes negative as the moment of Gd^3+^ ion is increased while *M*_Cr_^3+^ remains the same. As a consequence, *M*_Gd_^3+^ dominates over the magnetization corresponding to *M*_Cr_^3+^. A typical *M*_FC_ magnetization *vs.* temperature plot of Cr^3+^ and Gd^3+^ under 50 Oe at *T* > *T*_comp_ and *T* < *T*_comp_ is shown in Fig. 4(b). While with applying low field, we could observe a magnetization maxima in FC curve and magnetization reversal followed by spin reorientation transition, with application of high field (500 Oe), the rotation of moment of M_Gd_^3+^ ion takes place along the external field direction since the applied field is large enough to overcome the internal field generates by Cr^3+^ ions. Hence a positive magnetization is observed in whole temperature range. With varying Cr concentration, the applied field strength also changes which essentially evokes a positive magnetization in FC mode. For instance, in *x* = 0.1 and *x* = 0.2, the magnetization is always positive independent of applied field. However, in *x* = 0.3 while the FC magnetization is positive under 500 Oe, we do not observe positive magnetization upto 500 Oe for *x* = 0.4. Magnetization reversal has been reported in orthochromites, orthoferrites and orthovanadates.^45–47^ The *T*_SR_ is also observed at ∼4.8 K from (*M*_ZFC_ − *M*_FC_)/*M*_ZFC_ plot as in case of *x* = 0.3. The presence of *T*_SR_ could be ascribed to the interactions between different ion pairs such as Gd–Gd, Cr–Mn and Gd–Mn.

**Fig. 5:**
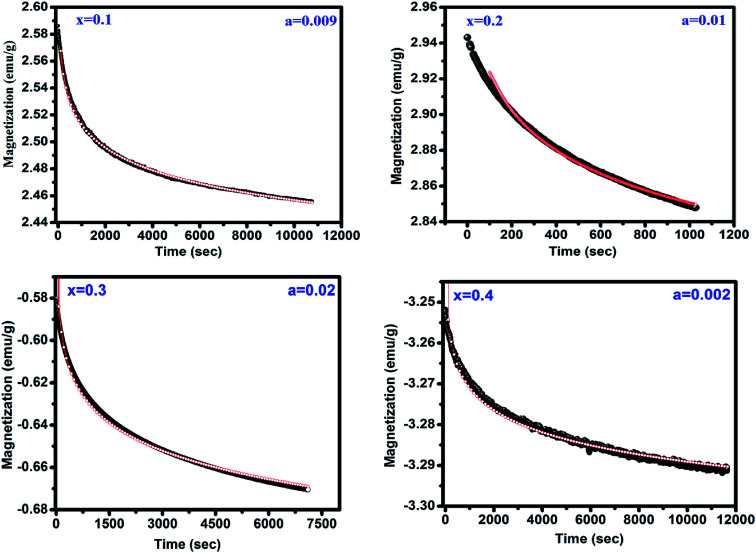
Magnetic relaxation after field cooling in 100 Oe. The red line shows the fitting to the power law function.

The peak *T*_P_ observed in *M vs. T* measurements is a possible fingerprint of the presence of spin-glass (SG) behavior. The presence of SG behavior has been validated after analyzing the time dependent remanant magnetization measurements for *x* = 0.1–0.4 shown in Fig. 5. This measurement is carried out by cooling sample from 300 to 15 K in under applied magnetic field of 100 Oe and then the field is removed. The magnetization is measured with varying time for 3 h. In general, the relaxation for SG behavior satisfies the power law, *M* (*t*) = *M*_0_*t*^−*b*^ where *M*_0_*i.e. M* at *t* = 0 known as initial magnetization and *b* is the decay parameter.^22^ It is clear from fitting of experimental data with above equation that the remanent magnetization satisfies the power law corroborating the SG like behavior in all samples.

**Fig. 6:**
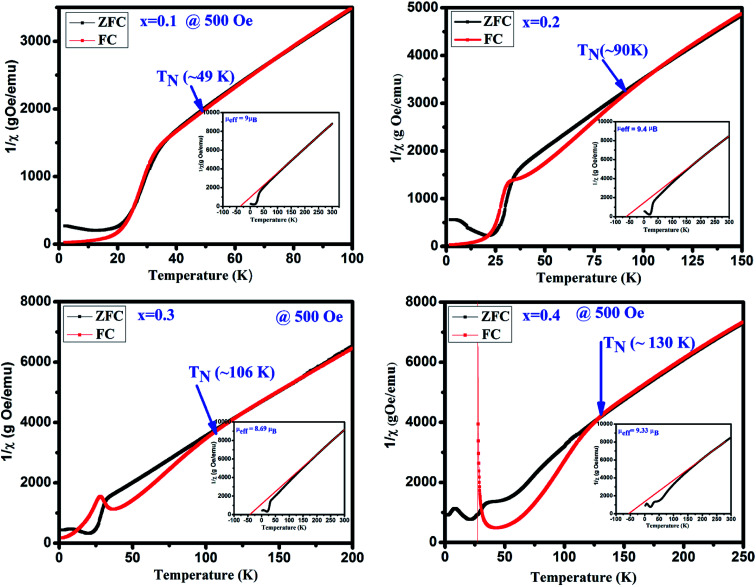
The inverse susceptibility 1/*χ* plots of ZFC and FC in the presence of 500 Oe and insects show Curie–Weiss law fitting of the GdMn_1−*x*_Cr_*x*_O_3_ (*x* = 0.1, 0.2, 0.3 and 0.4).

To determine the magnetic transition temperature, 1/*χ vs.* temperature curves are plotted for GdMn_1−*x*_Cr_*x*_O_3_ (*x* = 0.1–0.4) shown in Fig. 6. The antiferromagnetic Néel temperature (*T*_N_) is found to be increased from ∼49 and ∼130 K when *x* is varied from 0.1 to 0.4. The estimated values are much higher than that of GdMnO_3_ (*i.e. T*_N_ ∼ 42 K).^24^ It is interesting that as we dope Cr^3+^ in Mn^3+^ site, the *T*_N_ is increased. This may be understood in terms of dilution of the Mn–Mn interaction due to Cr–Mn and Cr–Cr exchange interactions. In addition, from 1/*χ vs.* temperature plots, the effective magnetic moment (*μ*_eff_) is calculated after fitting the data in paramagnetic region using Curie–Weiss law *i.e.* 1/*χ* = (*T* − *Θ*)/*C*, where *C* is Curie constant and *Θ* is Curie–Weiss temperature (insets of Fig. 6). The negative value of *Θ* indicates the antiferromagnetic coupling between the atoms in all samples. The *μ*_eff_ is estimated theoretically using the formula, *μ*_eff_ = [(*μ*_Gd_)^2^ + (1 − *x*) (*μ*_Mn_)^2^ + *x*(*μ*_Cr_)^2^]^1/2^. The theoretical values of *μ*_eff_ are found to be 9.25, 9.20, 9.15 and 9.10 *μ*_B_ for *x* = 0.1, 0.2, 0.3 and 0.4, respectively, which clearly shows a decreasing trend with increasing Cr concentration.

**Fig. 7:**
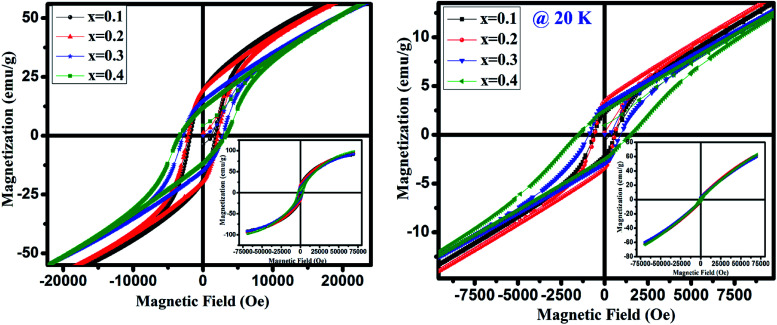
Magnetic field dependent magnetization at 4 K and 20 K of GdMn_1−*x*_Cr_*x*_O_3_ (*x* = 0.1, 0.2, 0.3 and 0.4).

Further we have recorded the magnetization (*M*) as a function of the external field (*H*) below *T*_N_*i.e.* at 20 and 4 K for GdMn_1−*x*_Cr_*x*_O_3_ (*x* = 0.1–0.4) in ZFC mode shown in Fig. 7. At 20 K, the magnetization with a slim loop increases linearly with increasing field which does not saturate upto 70 kOe (inset of Fig. 7). Further, with decreasing the temperature to 4 K, the area under the loop enhances and the loops are symmetrical along magnetic and magnetization axes, indicate the absence of exchange bias. After analyzing the hysteresis loops measured at 4 and 20 K, the calculated values of maximum magnetization (*M*_max_), coercivity (*H*_c_) and remanence (*M*_r_) are given in Table 3. One may note that *M*_max_ increases with increase in Cr concentration except at *x* = 0.3 corroborates with *μ*_eff_ calculated from Curie–Weiss fitting and is in contrast with the theoretical value of *μ*_eff_. The increase in *M*_max_ thus supports the presence of Mn^4+^ and oxygen vacancies. It is presumed that as the concentration of Mn^4+^ decreases compared to Mn^3+^ with increase in Cr concentration, magnetization increases as observed in the present case. Generally, the magnetization hysteresis loop and its linear increasing nature at high field appear due to the weak ferromagnetic ordering which primarily induced by deviation of the collinearity of the moments in an antiferromagnet. Mao *et al.* discuss a similar behavior of magnetization as a function of external field in case of YFe_0.5_Cr_0.5_O_3_.^48^

**Table 2:** Fitting parameters for *M*–*T* curves recorded in the FC mode for GdMn_1−*x*_Fe_*x*_O_3_ (*x* = 0.3 and 0.4)

Composition	External field	*M*_Cr_ (emu g^−1^)	*H*_I_ (Oe)	*Θ*
*x* = 0.3	50 Oe	0.98	−61.32	−51.24
	100 Oe	1.88	−121.50	−50.89
*x* = 0.4	50 Oe	7.56	−188.42	−28.73
	100 Oe	11.40	−278.16	−34.54
	500 Oe	12.74	−642.79	−49.4

**Table 3:** Maximum magnetization (*M*_max_), remanence (*M*_r_) and coercivity (*H*_c_) for GdMn_1−*x*_Cr_*x*_O_3_ (*x* = 0.1, 0.2, 0.3 and 0.4) samples at 4 K and 20 K temperature

Temperature	*M*_max_@7 kOe (emu g^−1^)				*H*_C_ (Oe)				*M*_r_ (emu g^−1^)			
	*x* = 0.1	*x* = 0.2	*x* = 0.3	*x* = 0.4	*x* = 0.1	*x* = 0.2	*x* = 0.3	*x* = 0.4	*x* = 0.1	*x* = 0.2	*x* = 0.3	*x* = 0.4
4 K	90	95	92	98	1815	2158	2838	3232	19.3	19	14	12
20 K	60	63	60	63	619	593	924	1556	2.2	3	3	2.3

**Fig. 8:**
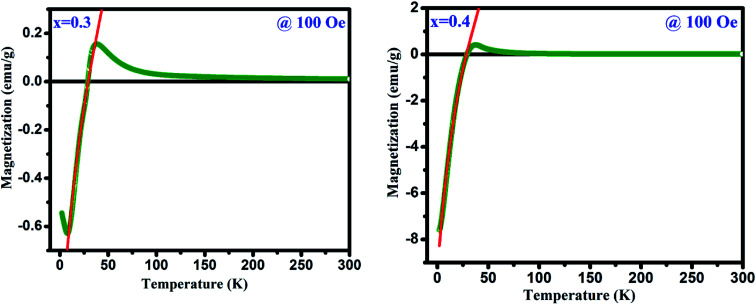
Magnetization curves at 100 Oe applied field in FC mode (the red line shows fitting with eqn (2)).

**Fig. 9:**
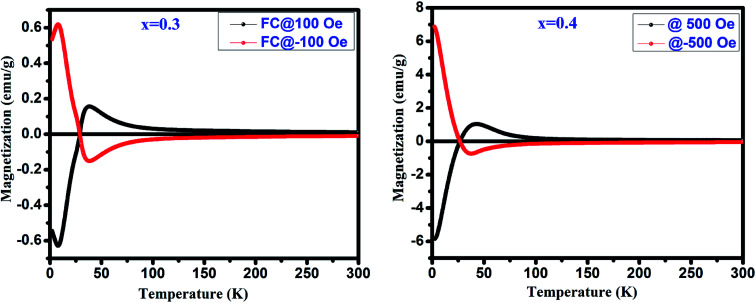
Mirror like behavior in FC mode for GdMn_1−*x*_Cr_*x*_O_3_ (*x* = 0.3 and 0.4).

The magnetization reversal has been further investigated by fitting the FC magnetization curves employing following equation:2
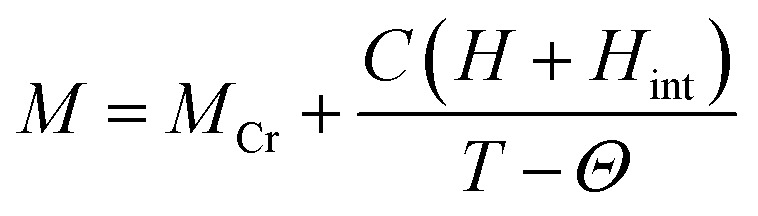
where *M*, *M*_Cr_, *C*, *H*, *H*_int_ and *Θ* is called as the total magnetization, magnetization due to the Cr^3+^ ions, Curie constant, applied magnetic field, internal magnetic field from Cr^3+^ ions, and Weiss temperature, respectively. The fittings of *M*_FC_ at 100 Oe are shown in Fig. 8 and the estimated parameters are tabulated in Table 2 under the applied fields of 50, 100 and 500 Oe (figures are not shown). From Table 2, it is noteworthy to mention that *H*_int_ (due to Cr^3+^ ions) and the applied magnetic field (*H*) are in opposite direction to each other which changes with increasing *H*. The estimated enhancement in *H*_int_ and *M*_Cr_ values are attributed to the increase in strengthening of AFM ordering under an applied field.^28^ Biswas *et al.* have also found same behavior in the case of Gd_0.7_Ca_0.3_Mn_1−*x*_Cr_*x*_O_3_ (*x* = 0.0–0.5).^49^ Moreover, under low applied magnetic field, the canted Cr^3+^ and Gd^3+^ ions interact antiferromagnetically giving rise to the negative value of *Θ* in the present system. Further, we have investigated the antiferromagnetic ordering of net magnetic moments applying external magnetic field below *T*_comp_. Explicitly, a mirror-like behavior observed at ±100 Oe for *x* = 0.3 and at ±500 Oe for *x* = 0.4 is shown in Fig. 9.

**Fig. 10:**
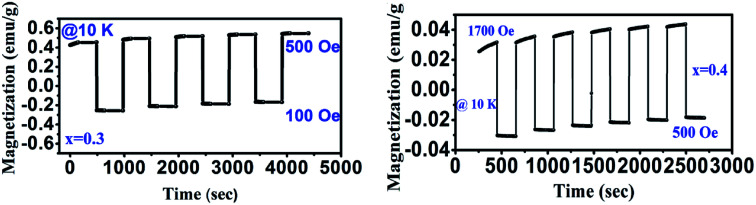
Magnetic switching behavior of GdMn_1−*x*_Cr_*x*_O_3_ (*x* = 0.3 and 0.4) at different field.

Owing to the existence of characteristic features like magnetic reversal, Cr doped GdMnO_3_ (*x* = 0.3 and *x* = 0.4) has been explored for distinct magnetic switching effect. At 100 Oe, the sample is cooled down to 10 K in FC mode and magnetic switching measurements are recorded shown in Fig. 10. First, the magnetization is measured for 180 s at 10 K. Then, the magnetic field is quickly increased to 500 Oe and 1700 for *x* = 0.3 and 0.4, respectively followed by measuring the magnetization for constant time interval of 180 s. Apparently, these samples exhibit a promising magnetic switching effect. The measurement cycles are repeated for several times to examine the switching reproducibility which indicated reversible and continuous switching of the magnetization over cycles. The switching between the positive and negative magnetization states can be triggered through changing the magnitude of the field with fixed field direction. Moreover, this can be tuned in a predictable way. The magnetic switching effects driven by external magnetic field render promising application of these materials in magnetic data storage and magnetic switching based nonvolatile magnetic memory.

## Conclusions

In summary, we systematically examined the structural evolution and rich sequence of magnetic transitions in Cr doped GdMnO_3_ synthesized using sol–gel method. As Cr concentration is increased from 0.1 to 0.4, we observed the structural transformation from O′ to O type orthorhombic one along with the reduction in lattice volume. The decrease in lattice volume was due to the smaller ionic radius of Cr^3+^ ion compared to Mn^3+^. The structural transformation was manifested by the reduction in J–T distortion factor estimated by the bond length obtained from Rietveld refinement. Raman spectra supported the observed reduction in J–T distortion factor as reflected showing decrease in the intensity of asymmetric stretching bonds at 487 and 610 cm^−1^. Further, the emission peak at ∼286 nm in PL spectra disappeared indicating decrease in J–T distortion factor. PL study demonstrated the emission spectra related to Mn^4+^ energy levels which improved after incorporating 10 at% of Cr^3+^. Magnetic measurements showed an increase in *T*_N_ from ∼42 K for *x* = 0 to ∼130 K when *x* reached 0.4. Besides magnetization reversal with spin reorientation and magnetic switching effect are also observed as *x* reached 0.3. These materials can be used in magnetic switching, magneto caloric and spintronics devices.

## Conflicts of interest

There are no conflicts to declare.

## Supplementary Material
